# Neuroprotection by Neurotropin through Crosstalk of Neurotrophic and Innate Immune Receptors in PC12 Cells

**DOI:** 10.3390/ijms21186456

**Published:** 2020-09-04

**Authors:** Yu Fukuda, Kazuki Nakajima, Tatsuro Mutoh

**Affiliations:** 1 Department of Neurology and Neuroscience, Fujita Health University Hospital, Toyoake, Aichi 470-1192, Japan; y-fukuda@nippon-zoki.co.jp; 2 Nippon Zoki Pharmaceutical Co., Ltd., Osaka 564-0052, Japan; 3 Center for joint research facilities support, Research Promotion and Support Headquarters, Fujita Health University School of Medicine, Toyoake, Aichi 470-1192, Japan; k-nakaji@fujita-hu.ac.jp

**Keywords:** Neurotropin, lipid rafts, GM1 ganglioside (GM1), TrkA tyrosine kinase, Toll-like receptor4 (TLR4), Fyn

## Abstract

Infected or damaged tissues release multiple “alert” molecules such as alarmins and damage-associated molecular patterns (DAMPs) that are recognized by innate immune receptors, and induce tissue inflammation, regeneration, and repair. Recently, an extract from inflamed rabbit skin inoculated with vaccinia virus (Neurotropin^®^, NTP) was found to induce infarct tolerance in mice receiving permanent ischemic attack to the middle cerebral artery. Likewise, we report herein that NTP prevented the neurite retraction in PC12 cells by nerve growth factor (NGF) deprivation. This effect was accompanied by interaction of Fyn with high-affinity NGF receptor TrkA. Sucrose density gradient subcellular fractionation of NTP-treated cells showed heretofore unidentified membrane fractions with a high-buoyant density containing Trk, B subunit of cholera toxin-bound ganglioside, flotillin-1 and Fyn. Additionally, these new membrane fractions also contained Toll-like receptor 4 (TLR4). Inhibition of TLR4 function by TAK-242 prevented the formation of these unidentified membrane fractions and suppressed neuroprotection by NTP. These observations indicate that NTP controls TrkA-mediated signaling through the formation of clusters of new membrane microdomains, thus providing a platform for crosstalk between neurotrophic and innate immune receptors. Neuroprotective mechanisms through the interaction with innate immune systems may provide novel mechanism for neuroprotection.

## 1. Introduction

Neurotrophins control survival and differentiation of neurons through plasma membrane high-affinity receptor tropomyosin-related kinase (Trk) [1,2]. Each neurotrophin, nerve growth factor (NGF), brain-derived neurotrophic factor (BDNF), neurotrophin-3 (NT-3), and NT-4, is capable of binding to a specific Trk receptor tyrosine kinase (TrkA, TrkB, and TrkC) and to the p75 low-affinity NGF receptor [3,4]. Initiation of neurotrophin signaling is strictly controlled by sequence of multiple events occurring on Trk molecules, which include interaction with GM1 ganglioside [5], formation of homodimers [6], autophosphorylation of tyrosine residues [1], and a spatial assembly with downstream adaptor molecules [7]. Moreover, Trks are known to localize in membrane microdomains enriched with cholesterol and sphingolipids [8,9,10,11], often referred to as lipid rafts, which are now recognized as important subcellular domains for various cellular signaling pathways by compartmentalizing particular transmembrane receptors with adaptors and other signaling molecules [12,13]. Lipid rafts for TrkB signaling are formed transiently and dynamically upon binding of its ligand BDNF [14] and are positively regulated by Fyn, a member of the Src-family kinases residing in lipid rafts [15].

A nonprotein extract of inflamed rabbit skin inoculated with vaccinia virus, designated Neurotropin^®^ (NTP), is prescribed widely in Japan and China for the treatment of chronic pain and other various neurological disorders. In vivo, NTP has demonstrated neuroprotective and anti-inflammatory activity in animal models of Down’s syndrome [16], Alzheimer’s disease (APP/PS1) [17], permanent middle cerebral artery occlusion [18], hypoxic ischemic brain injury [19], chemotherapy-induced peripheral neuropathy [20,21], and peripheral nerve injuries [22,23]. However, the precise molecular mechanisms underlying these pharmacological actions currently remain to be elucidated. We recently reported that expressions of neurotrophins were enhanced in human neuroblastoma SH-SY5Y cells by NTP through activation of autocrine pathways depending on TrkB activity [16]. In subsequent studies using rat pheochromocytoma PC12 cells overexpressing Trk (PCtrk cells) [24], NTP was shown to facilitate the TrkA-mediated neurotrophin signaling pathway [25].

In the present study, we demonstrate a role for innate immune receptor, toll-like receptor 4 (TLR4) in the neuroprotective actions of NTP. Treatment of PCtrk cells with NTP resulted in the formation of high-buoyant density, unknown lipid rafts-like membrane fractions where cholesterol, B subunit of cholera toxin-bound gangliosides, Trk, Fyn, flotillin-1, and TLR4 were co-segregated, which were dissociable by TLR4 inhibition. These observations provide a novel function of lipid rafts-like microdomains that bridge neurotrophic and innate immune systems whereby neuronal cells are preserved even under insufficient conditions.

## 2. Results

### 2.1. NTP Protects Differentiated PCtrk Cells from NGF Deprivation

**Figure 1:**
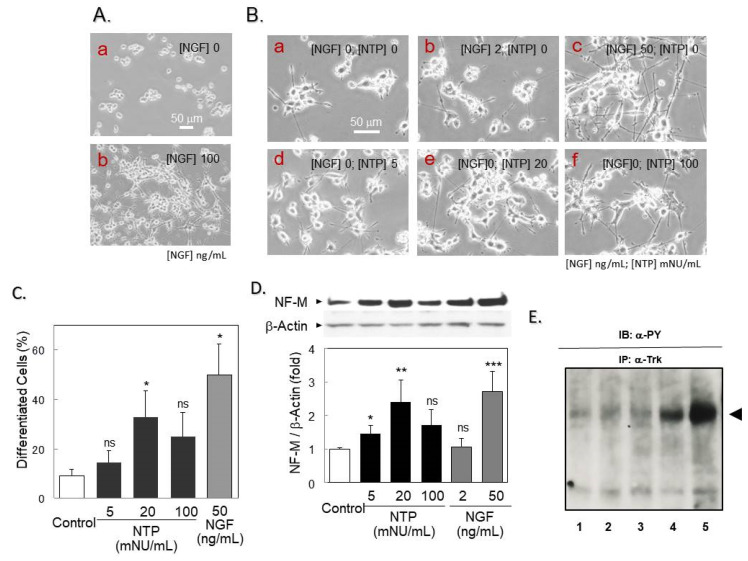
Neurotropin (NTP) protected differentiated PC12 cells overexpressing Trk (PCtrk) cells from Nerve growth factor (NGF) deprivation. (**A**) NGF-mediated morphological differentiation. PCtrk cells (3000 cells in ϕ3-cm dish) were cultured for 24 h in the absence (**a**) or presence (**b**) of NGF (100 ng/mL). Phase-contrast micrographs were captured for typical areas of cultures. (Bar = 50 μm in length). (**B**) Effect of NTP on neurite retraction induced by NGF deprivation. Differentiated PCtrk cells were gently washed with PBS and incubated for additional 30 h in serum-free DMEM in the presence (**b**, 2 ng/mL; **c**, 50 ng/mL) or absence (**a**, **d**-**f**) of NGF. NTP was added to the medium at 5 mNU (**d**), 20 mNU (**e**) or 100 mNU (**f**) per mL. The degree of differentiation of cells in cultures supplemented with saline (Control), NTP (5, 20, or 100 mNU/mL), or NGF (50 ng/mL) was quantified as described in Materials and Methods, and summarized in (**C**). Data represent means and standard deviations (SD) of three independent cultures. * *p* < 0.05 vs. saline-treated controls. ns, not significant. (**D**) Neurofilament M (NF-M) expression. Cell lysates of PCtrk cells (1 × 10^5^ cells, 32 h) were subjected to Western blotting against NF-M (160 KDa) and β-actin (internal control, 45 KDa) as described in Materials and Methods (upper panel, typical blotting images). Data represent mean and SD of the intensity ratios of NF-M to β-actin from three independent cultures. * *p* < 0.05, ** *p* < 0.01 and *** *p* < 0.001 vs. saline-treated controls (open bar); ns, not significant (two-sided *t*-test). (**E**) Autophosphorylation status of TrkA during de-differentiation by the withdrawal of NGF. The cells were pretreated with NGF for 24 h and then further treated for 30 h either with NGF or NTP or untreated in the same way as (**B**). The TrkA protein was immunoprecipitated with anti-TrkA antibody and subjected to the Western blotting probed with anti-phosphotyrosine antibody. Note that there was no significant difference in the autophosphorylation status in each treatment. 1: untreated cells; 2: NGF-treated; 3: NTP-treated; 4: naïve cells; 5: NGF-treated cells (50 ng/mL, 5 min).

In PCtrk cells, NGF dramatically promoted neurite outgrowth, a morphological marker of cellular differentiation, within 24 h (Figure 1A, Panel b). The cell population possessing neurites that extended larger than the cell body diameter was 82.5 ± 15.2% (mean ± SD). Withdrawal of NGF from culture medium resulted in significant neurite retraction within 30 h (Figure 1B, Panel a). A continuous supply of NGF restored neurite extensions in a concentration-dependent manner (Figure 1B, Panels b and c). In the absence of NGF, a gradual loss of neurites was observed within next 48 h, and cell viability dramatically dropped by 72 h based on trypan blue dye exclusion assay. Such observations indicated that neurite extension and retraction are reversible processes dependent on NGF. Even when cells were deprived of neurotrophic support, NTP prevented this morphological dedifferentiation in a dose-limited manner (Figure 1B, Panels d,f). Maximal preservation of neurites was observed at a dose of 20 mNU/mL, and the effect was partly diminished at a higher dosage (100 mNU/mL; Figure 1C). To confirm this effect biochemically, intracellular expression of neurofilament M (NF-M), a major constituent of neurites, was evaluated by Western blotting. Even without exogenous NGF, expression of NF-M was strongly maintained by NTP (20 mNU/mL; Figure 1D). Activation status of TrkA was affected neither by NGF nor NTP during chronic phase of exposure (Figure 1E). Taken together, these data suggest that NTP protected PCtrk cells from neurite retraction induced by NGF deprivation. Moreover, a similar neuroprotection by NTP was observed in parental PC12 cells, indicating that this action is not strain-specific (data not shown).

### 2.2. NTP Accelerates Association of Fyn with Trk

**Figure 2:**
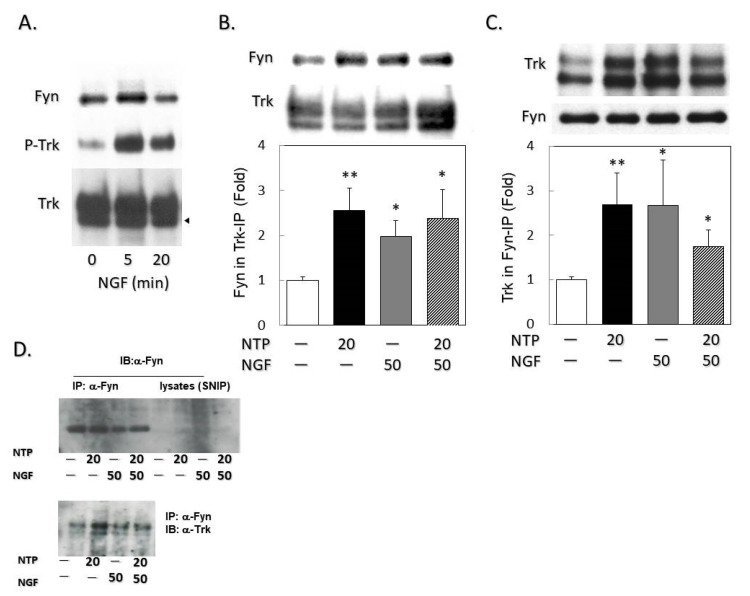
NTP enhanced the interaction of Trk with Fyn. (**A**) NGF-dependent association of Fyn with Trk. Cell lysates from PCtrk cells (5 × 10^5^ cells) treated with NGF (50 ng/mL) for indicated periods (0, 5, 20 min) were immunoprecipitated with α-Trk (clone C-14) as described in Materials and Methods. Precipitates were examined by Western blotting against Fyn (59 KDa), phosphotyrosine (P-Trk, 140 KDa), or Trk (140 KDa). α -Trk immunoblotting demonstrated split bands which represent full and truncated (arrowhead) forms of Trk in PCtrk cells. Representative images are shown. (**B**) Fyn was detected in α-Trk immunoprecipitates. Trk was immunoprecipitated from cell lysates of PCtrk cells (5 × 10^5^ cells) treated with NTP (20 mNU/mL, 3 h) and/or NGF (50 ng/mL, 5 min) as indicated, and subjected to Western blotting against Fyn or Trk. Data represent the band intensity of the Fyn/Trk ratio in immunoprecipitates (control = 1). (**C**) Trk detected in α-Fyn immunoprecipitates was similarly examined by Western blotting. Data represent the band intensity of Trk/Fyn ratio in immunoprecipitates (control = 1). All data are expressed as means and SDs of three independent cultures. * *p* < 0.05, and ** *p* < 0.01 vs. saline-treated controls (open bar); ns, not significant (two-sided *t*-test). Representative images are shown in upper panels. (**D**) To examine the enrichment of Fyn protein in respective α-Fyn immunoprecipitates, total cell-free supernatants after immunoprecipitation (SNIP) were also subjected to Western blotting. Note that almost equal Fyn protein was immunoprecipitated from lysates prepared from respective treatment. These examinations were performed four times with four different preparations with essentially identical result. Typical figure was shown.

Fyn is a crucial regulator of neurotrophin signaling by modulating translocation of Trk receptors into lipid rafts [26]. NGF treatment (50 ng/mL) caused autophosphorylation of TrkA with a peak at 5 min, which was accompanied by molecular interaction with Fyn (Figure 2A). Treatment with NTP (20 mNU/mL, 3 h) similarly enhanced the association of Trk with Fyn, even in the absence of exogenously added NGF (Figure 2B–D). These data suggest that NTP controls the recruitment of Trk to the proximity of Fyn. Moreover, in order to show the enrichment of Fyn protein in respective α-Fyn immunoprecipitates, total cell-free supernatants after immunoprecipitation (SNIP) were also subjected to Western blotting. We confirmed Fyn-enrichment in α-Fyn-immunoprecipitates from each treatment group (Figure 2D). 

### 2.3. High-Density Lipid Rafts-Like Membrane Fractions in NTP-treated PCtrk Cells

**Figure 3:**
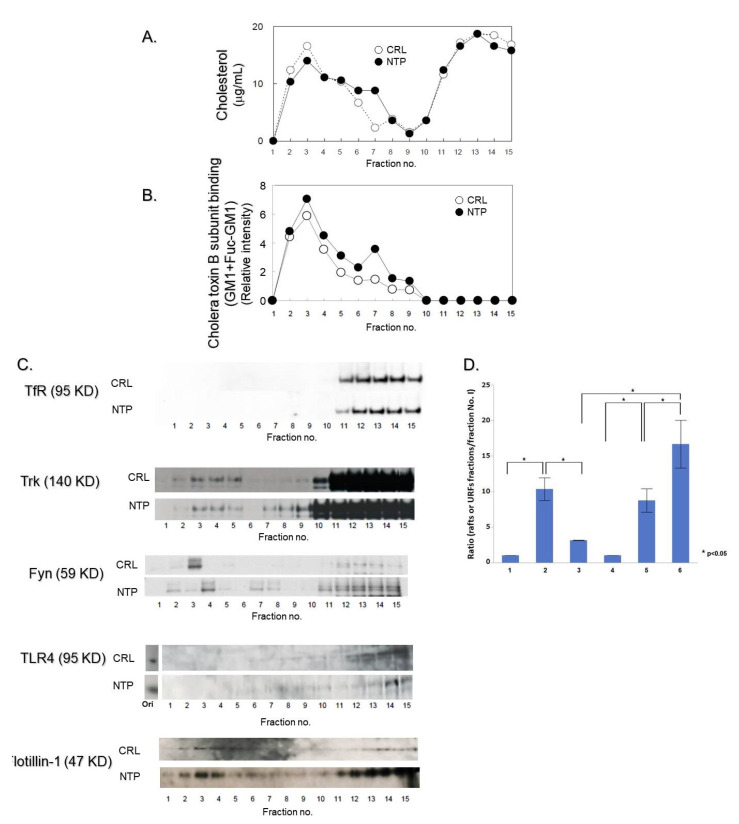
Subcellular distribution of lipid rafts in PCtrk cells treated with NTP. PCtrk cells (5 × 10^6^ cells) were treated with 20 mNU/mL NTP (closed symbols, or NTP) or saline (open symbols, or CRL) for 3 h, lysed in 0.5% Triton X-100-containing TNE buffer and centrifuged in sucrose density gradients to isolate lipid rafts, as described in Materials and Methods. Representative data are shown. (**A**) Cholesterol concentrations determined by Amplex Red Cholesterol Assay Kit. (**B**) GM1 ± fucosyl-GM1 concentrations determined by dot blot analysis using cholera toxin subunit B subunit- conjugated with horseradish peroxidase. (**C**) Distribution of transferrin receptor (TfR, 95 KDa), Trk (140 KDa), Fyn (59 KDa), Toll-like receptor TLR4 (95 KDa), and flotillin-1 (47 KDa) determined by Western blotting. Detergent-insoluble typical lipid rafts were fractionated in this condition into fractions 2–5. Note that NTP treatment formed novel membrane fractions (no. 6–8) with high-buoyant density (unidentified raft-like fractions, URFs). (D) Statistical analysis of the Trk protein distribution in lipid rafts and URFs fraction. We measured the densities of each band corresponding to Trk in fractions by ImageJ software (ver. 1.51; NIH, USA; RRID: SCR_003070). Then, we calculated the ratio of densities of each fraction corresponding to rafts (fraction No. 2–5; columns 2 and 5, n = 4) and URFs (fraction No. 6–8; columns 3 and 6, n = 4) against that of fraction No. 1 (columns 1 and 4; this fraction did not contain Trk protein but exhibit background densities, n = 4) and further calculated the mean ratio ± SE of rafts fractions and of URFs. These data were subjected to the statistical analysis (Wilcoxon ranked sum test) * *p* < 0.05. We confirmed that Trk protein is definitely present in lipids rafts fractions in control (columns 2) and NTP-treated (column 5) cells and NTP-treatment caused a statistically significant redistribution of the Trk protein into URFs fractions (column 6) than that of control cells (column 3).

We next examined the effects of NTP on lipid rafts formation in PCtrk cells. Lipid rafts are defined as detergent-insoluble glycosphingolipids/cholesterol-rich membrane microdomains characterized physicochemically by their insolubility in nonionic detergents and low buoyant density. Following lysis with Triton X-100 for 30 min at 4 °C and ultracentrifugation in discontinuous sucrose density gradients ultracentrifugation, fractions were collected and analyzed by Western blotting (Figure 3). Lipid rafts were recovered mainly in fractions no. 2–5 that were enriched in lipid markers, cholesterol and B subunit of cholera toxin-bound ganglioside (GM1 ± fucosyl-GM1 ganglioside) as well as flotillin-1 protein (Figure 3A,B, open circles). Non-rafts membranes containing transferrin receptor (TfR) stayed in the bottom fractions of the gradients. Although Trk was observed in the bottom fractions, it was also definitely recovered in the lipid rafts fractions in these cells (2.83 ± 0745% of total Trk protein (mean ± SEM, n = 4) (Figure 3C, D, CRL). By contrast, incubation with NTP (20 mNU/mL, 37 °C, 3 h) significantly induced the formation of fractions containing unknown membrane components (fractions no. 6–8), that resembled lipid rafts because of the enrichment of raft markers such as cholesterol, B subunit of cholera toxin-bound gangliosides (Figure 3A,B, closed circles) and flotillin-1 (Figure 3C), but they seemed to be slowly migrating upward in the gradients (unidentified rafts-like fractions, URFs) than compared with those of controls. A definite amount of TrkA was also distributed in these fractions (Figure 3C,D, NTP). In addition, the most prominent effect of NTP was observed in the distribution of Fyn. A considerable amount of Fyn was distributed in the URFs in NTP-treated, but not in control PCtrk cells. By contrast, NTP did not affect the main distribution of cholesterol and GM1 ± fucosyl-GM1 into lipid rafts fractions.

### 2.4. Role of TLR4 in Neuroprotection by NTP

We have previously shown that NTP accelerates NGF signaling which accompanies enhanced association of Trk with GM1 and possibly fucosyl-GM1 [25], an essential cofactor for Trk tyrosine kinase [5]. Because GM1 is also known as a modulator of innate immune receptor TLR4 [27], we examined whether TLR4 has a role in the neuroprotective effects of NTP. As shown in Figure 3C, TLR4 was uniformly distributed in the bottom, non-raft fractions in PCtrk cells (CRL). By contrast, NTP treatment resulted in an accumulation of TLR4 within the URFs, albeit not in typical lipid rafts fractions.

**Figure 4:**
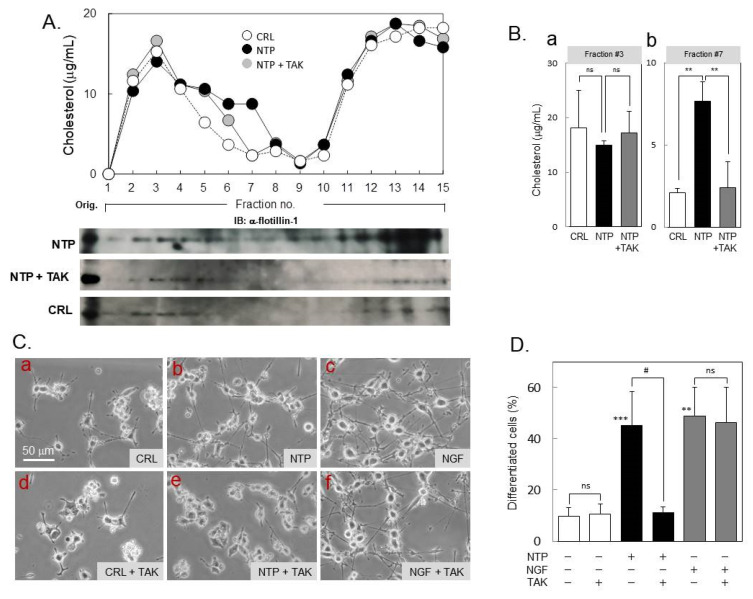
Role of Toll-like receptor 4 (TLR4) in the neuroprotection by NTP. (**A**) Effect of TAK-242 on NTP-induced formation of URFs. PCtrk cells (5 × 10^6^ cells) were treated in serum-free DMEM containing saline control (open symbols, or CRL), NTP (20 mNU/mL, closed symbols), or NTP and TAK-242 (10 nM, gray symbols, or TAK) for 3 h, lysed in 0.5% Triton X-100-containing TNE buffer and centrifuged in sucrose density gradients as described in Materials and Methods. Cholesterol levels in each fraction were quantified by Amplex Red Cholesterol Assay Kit to confirm peaks of the lipid rafts and URFs. Representative data are shown. Moreover, we also analyzed a rafts-marker protein, flotillin-1 distribution in sucrose density gradient fractionation of the cells treated with each treatment. NTP-treatment caused new emergence of flotillin-1 in URFs and it was abolished by TAK-242 treatment. (**B**) Cholesterol contents in the peak fraction of typical lipid rafts (Panel **a**, fraction no.3) and of URFs (Panel **b**, fraction no. 7) are summarized as mean and SD of three independent experiments. ns, not significant; ** *p* < 0.01 between groups (ANOVA). (**C**) Effect of TAK-242 on prevention of neurite retraction by NTP. PCtrk cells (3000 cells in ϕ3-cm dish) were differentiated by 100 ng/mL of NGF for 24 h, and treated for an additional 30 h in serum-free DMEM containing saline (**a**, **d**, CRL), NTP (**b**, **e**, 20 mNU/mL), NGF (**c**, **f**, 50 ng/mL), and/or TAK-242 (**d**-**f**, 10 nM). Representative phase-contrast micrographs were captured for typical areas of cultures. (Bar = 50 μm in length). (**D**) Degree of differentiated cells (%) in cultures was counted as described in Materials and Methods and is summarized as mean and SD of three independent cultures. ** *p* < 0.01; *** *p* < 0.001 vs. CRL. ns, not significant; # *p* < 0.05 between groups (ANOVA).

To assess the functionality of TLR4 in the neuroprotective actions of NTP, we tested the effects of TAK-242, a selective inhibitor that interferes with interactions between TLR4 and its adaptor molecules [28]. In sucrose density gradient fractions, cholesterol levels in URFs (fraction no. 7) formed by NTP treatment were significantly diminished by 10 nM of TAK-242 (Figure 4A,B, NTP ± TAK), indicating dissociation of URFs. Total cellular cholesterol levels were not significantly different between CRL, NTP, and NTP ± TAK groups (2.09 ± 0.37, 2.16 ± 0.16, and 2.29 ± 0.38 μg per mg protein, respectively). Moreover, NTP caused rafts-marker protein, flotillin-1 redistribution in URFs as well as typical rafts fractions, whereas NTP ± TAK treatment cancelled the distribution of flotillin-1 in URFs (Figure 4A). These data suggest that TLR4 activity is required for the formation of URFs with NTP treatment. In addition, TAK-242 strongly blocked neuroprotective effects of NTP, but not those of NGF, on PCtrk cells (Figure 4C,D). Thus, TLR4 activation may be involved in the neuroprotective actions of NTP, perhaps through formation of URFs where intermolecular crosstalk between Trk and TLR4 occurs.

## 3. Discussion

Neuritogenic actions by NTP had been reported for the first time by Morita et al. [29], where NTP enhances neurite outgrowth of PC12 cells. In the present study, NTP prevented neurite retraction in PCtrk cells under conditions of NGF-deprived states (Figure 1). In a placebo-controlled, double-blinded clinical trial on acute ischemic stroke, intravenous administration of NTP for 15 days was effective on the survival, infarct size, edema, and neurological symptoms of the patients [30,31]. Consistent observations were made recently in a mouse model of hypoxic ischemic brain injury supplemented by intraperitoneal injection of NTP, in which an associated significant decline in mRNA expressions of pro-inflammatory cytokines such as interleukin (IL)-6, IL-1β, and TNFα was demonstrated [19].

Neurotrophin signaling mediated by TrkA tyrosine kinase is necessary for the maintenance of the autonomic, peripheral, and central nervous systems. In the previous studies, we have shown the involvement of TrkA-mediated neurotrophin signaling in neuroprotective mechanisms by NTP in PCtrk cells [16,25]. Moreover, GM1 deficient cells or GM1 depleted cells with D-PDMP did not exhibit normal response of TrkA against its ligand, NGF [32,33]. 

In cultured cortical and hippocampal neurons, ligand-activated transient internalization into intracellular membranes and localization of Trk molecules into lipid rafts are crucially dependent on the association with the Src-family kinase Fyn [15]. Similarly, Fyn has been implicated in the translocation of membrane proteins, such as extracellular matrix heparin sulfate proteoglycan agrin [34], protein-tyrosine phosphatase RPTPα [35], and β-amyloid oligomers [36] into lipid rafts. In NGF signaling, Fyn-mediated recruitment of TrkA into detergent-resistant membranous compartments is thought to be the rate-limiting, temperature-sensitive process that may require transient internalization of TrkA as reported for TrkB [15]. In the present study, NTP treatment resulted in the association of Trk with Fyn (Figure 2), and these two molecules as well as TLR4 and flotillin-1 were recovered in the URFs in NTP-treated PCtrk cells (Figure 3C). Based on these observations, we extrapolate the involvement of Fyn in redistributing TrkA into URFs.

Innate immune pattern-recognition receptors (PRRs) such as TLRs, Nod-like receptors, RIG-like receptors, AIM2-like receptors and C-type lectin receptors, are capable of recognizing wide-array of molecules derived from pathogens (alarmins) and host damages (DAMPs) [37]. PRR recognition initiates a pro-inflammatory response to remove damaged cell debris from the affected region, which subsequently allows resolution of inflammation and regeneration of the tissues. Neuroinflammation, however, is often protracted under either sterile or infectious inflammatory diseases of the central nervous system (CNS) and may therefore contribute to neurodegeneration. TLR4 is one of the PRRs expressed in neuronal cells and microglia [38,39] that can detect and respond to alarmins/DAMPs, thus mediating microglial activation throughout the CNS [40]. In the present study, we have shown NTP-induced co-localization of TLR4 and TrkA in URFs (Figure 3). Similarly, the translocation of TLR4 into lipid rafts occurs in activated macrophages upon stimulation with its specific ligand, lipopolysaccharides (LPS) [41]. This has led to the concept that TLR activation requires the formation of “supramolecular activation clusters” [42] within membranes, proposing a new platform that brings receptor molecules close together, allowing their activation and signal transduction. Because the TLR4 inhibitor TAK-242 prevented formation of the URFs as well as the neuroprotective actions by NTP (Figure 4), the URFs are likely to construct signaling clusters specialized for TrkA-mediated neuroprotection. In agreement with our present observations on the patterns of URFs in sucrose density gradients, it was suggested that lipid rafts favor the increased density of lipid-associated adaptor molecules at the site of TLR4 clustering and signaling [43]. 

Gangliosides are sphingolipids that play a regulatory role in TLR4 translocation, spatial receptor-adaptor clustering in lipid rafts and receptor function. Recruitment of TLR4 and the cellular response upon LPS stimulation decreases in mutants that are deficient in sphingomyelin synthesis [44,45] or deficient in serine palmitoyl-CoA transferase, the key enzyme of de novo biosynthesis of sphingolipids [46]. In PC12 cells, LPS-induced lipid-rafts translocation of TLR4 is blocked by exogenous sphingolipids such as GM1 and GD1a gangliosides [47], leading to cellular evasion from LPS-induced toxicity. For the neuroprotective action of NTP, redistribution of TLR4 to URFs seems essential (Figure 3 and Figure 4). These URFs may serve as a platform reversibly formed for crosstalk between neurotrophic and innate immune systems, enabling efficient and lean TrkA-mediated NGF signaling [25]. Thus, GM1 and possibly fuc-GM1 may regulate neuronal cell fate by switching the subcellular localization of TLR4 in response to surrounding environmental signals. Although the existence of regulatory sphingolipids in NTP ingredients may partially explain its neuroprotective effects, an all-inclusive lipid analysis by HPLC failed to detect any sphingolipids or characteristic fatty acids patterns, whereas some phospholipids (e.g., phosphatidylcholine, phosphatidylserine and phosphatidylethanolamine) and neutral lipids (e.g., diacylglycerol and triacylglycerol) are present in trace amounts (data not shown).

In C57BL/6J mice with surgical occlusion at the left middle cerebral artery, repeated prophylactic oral administration of NTP (0.27 NU/kg/day, 3 weeks) induces significant reduction in infarcted lesion volume, brain edema, and neurological deficits [18]. Similar prophylactic neuroprotection has been reported in mice with administration of low-dose LPS [48,49,50,51,52] as well as ligands for other TLRs (i.e., TLR2, TLR3, TLR7, TLR8, and TLR9) as reviewed by Wang et al. [53]. Because TLR4 knockout largely diminishes the prophylactic effects of low-dose LPS, TLR4 is thought to be involved in establishing tolerance against infarct expansion [51]. NTP is manufactured from inflammatory rabbit cutaneous extracts after viral infection. Although substances with molecular weights exceeding 3000 are removed in the manufacturing processes, small compounds derived from inflamed tissue that are able to induce an innate immune response may be contained in NTP. Further analysis of the chemical components of URFs would guide precise molecular mechanisms linking Trk-mediated neurotrophic and TLR4-induced innate immune signaling occurring in these membrane microdomains.

## 4. Materials and Methods

### 4.1. Chemicals

All reagents were purchased from Sigma (St. Louis, MO, USA) unless otherwise stated. NTP was provided from Nippon Zoki Pharmaceutical Co., Ltd. (Osaka, Japan). The analgesic activity of NTP (expressed in Neurotropin unit, NU) is standardized by behavioral testing in rodents loaded with the “stress alteration of rhythm in environmental temperature” (SART), a repeated cold stress by which hypersensitivity to a noxious stimulus is produced [54]. NTP does not contain any detectable, known proteins such as NGF (by HPLC analysis). NTP was diluted in saline (Otsuka, Tokushima, Japan) as a vehicle. Protein concentration was determined by BCA protocol (Thermo Fisher Scientific, Waltham, MA, USA) with standard BSA solution (Thermo Fisher Scientific). TAK-242 was purchased from ChemScene (Monmouth Junction, NJ, USA) and dissolved in DMSO.

### 4.2. Cell Culture

PC12 cells overexpressing Trk (PCtrk cells; parental PC12 cell RRID: CVCL_0481) were constructed as described elsewhere [24]. The cells were grown in Dulbecco’s modified Eagle’s medium (DMEM, Invitrogen, Carlsbad, CA, USA) supplemented with 2 mM L-glutamine, 10% horse serum and 5% fetal bovine serum (BioWhittaker, Walkersville, MD, USA) on polystyrene culture flasks or dishes (Becton Dickinson and Company, Franklin Lakes, NJ, USA) at 37 °C in an humidified chamber supplied with 5% CO2. Expression of TrkA by PCtrk cells is almost 10-fold greater than parental PC12 cells cultured in normal DMEM. Cell viability was always >90% when assessed by staining dead cells with 0.4% trypan blue. Although there was a paper reporting no GM1 ganglioside but fucosyl-GM1 is present in PC 12 cells [55], our present PC12 cells contain both smaller but definite amount of GM1 and fucosyl-GM1 (data not shown) and therefore it might be dependent on the source of respective cell lines [5,24,32]. We should remind that CTB can recognize fucosyl-GM1 as well as GM1 and high-performance thin layer chromatography can separate each ganglioside. Fucosyl-GM1 standard was kindly donated by Prof. Masao Iwamori (Kindai University, Osaka, Japan).

### 4.3. Evaluation of Cell Differentiation

PCtrk cells (approximately 3000 cells) were allowed to adhere to six-well plate surfaces in DMEM and were stimulated with 100 ng/mL of 2.5S NGF (Millipore, Burlington, MA, USA) for 24 h. Differentiated cells were gently washed with phosphate-buffered saline (PBS) and incubated for additional 30 h in serum-free DMEM containing NTP or NGF or untreated. Cells possessing neurites longer than the greatest cell body diameter were defined as differentiated. For quantitation of the grade of differentiation, three independent cultures were assessed by image analysis with ImageJ software to calculate mean percentages of cellular differentiation. To elucidate the effect of TAK-242 on prevention of neurite retraction by NTP, PCtrk cells (3000 cells in ϕ3-cm dish) were differentiated by 100 ng/mL of NGF for 24 h, and treated for an additional 30 h in serum-free DMEM containing saline, NTP, NGF, and/or TAK-242 (10 nM). Representative phase-contrast micrographs were captured for typical areas of cultures. 

For detecting biochemical differentiation, PCtrk cells (1 × 10^5^) were lysed and homogenized at 4 °C in sodium dodecyl sulfate (SDS) sample buffer (58.3 mM Tris-HCl, pH 6.8, 1.7% SDS, 5% glycerol, 3.3% 2-mercaptoethanol, 0.002% bromophenol blue). Lysates were boiled and stored at −80 °C for Western blotting as described below. Blot intensities of NF-M and β-actin were determined by ImageJ software to calculate relative NF-M expression. The experiments to evaluate the molecular events induced by NTP were conducted on PCtrk cells differentiated for 24 h with NGF (50 ng/mL) in serum-free DMEM, subsequently subjected to saline, NGF, NTP or NTP-TAK-242 for 3 h at the concentrations specified in the text.

### 4.4. Immunoprecipitation and Immunoblotting

For cell-free supernatant preparation from PCtrk cells, medium was removed and cells were immediately washed with ice-cold PBS (4 °C), followed by treatment in lysis buffer (20 mM HEPES, pH 7.2, 1% Nonidet P-40, 10% glycerol, 50 mM NaF, 1 mM phenylmethylsulfonyl fluoride, 1 mM Na3VO4, 10 μg/mL leupeptin).

For immunoprecipitation, cellular supernatants after centrifugation at 10,000× *g* for 2 min at 4 °C were subjected to immunoprecipitation with antibodies against Trk (clone C-14; Santa Cruz Biotechnology, Santa Cruz, CA, USA; RRID: AB_632554; α-Trk), Fyn (sc-434; Santa Cruz Biotechnology; RRID: AB_627642; α-Fyn) and protein A-Sepharose conjugate at 4 °C overnight. After extensively washing, precipitates were eluted from the Sepharose beads by boiling in SDS sample buffer for 5 min. Eluates were subjected to SDS 5%–20% PAGE (ePAGEL; Atto Chemicals, Tokyo, Japan) and blotted onto a PVDF membrane (Immobilon-P; Millipore). Membranes were blocked for 1 h in Tris-buffered saline (TBS) containing 0.1% Tween 20 (TBS-T) with 3% nonfat milk. Incubations with the primary antibody and horseradish peroxidase (HRP)-coupled secondary antibody were performed for 1 h at room temperature in TBS-T. Immunoreactive bands were visualized by an enhanced chemiluminescence detection system (ECL Plus; GE Healthcare, Buckinghamshire, UK). Antibodies used for Western blotting were as follows: anti-Trk antibody (C-14), anti-Fyn antibody (sc-434), anti-phosphotyrosine antibody (clone 4G10; Millipore; RRID: AB_310802), anti-NF-M antibody (NA1216; Affiniti Research, Devon, UK; RRID: AB_10541956), anti-TfR(H68.4; Invitrogen; RRID: AB_86624), anti-TLR4 (sc-293072; Santa Cruz Biotechnology, Santa Cruz, CA, USA; PRID: AB_10611320; α-TLR4), anti-flotillin-1 antibody (610810; BD Transduction Laboratories, San Jose, CA, USA; RRID: AB_398189) and anti-β-actin antibody (clone 4967; Cell Signaling Technology, Danvers, MA, USA; RRID: AB_330288) for primary antibodies and anti-rabbit IgG (AP132P; Chemicon International, Temecula, CA, USA; RRID: AB_90264) and anti-mouse IgG (NA931; Amersham Bioscience, Buckinghamshire, UK; RRID: AB_772210) for secondary antibodies conjugated with HRP.

### 4.5. Sucrose Density Gradient Ultracentrifugation

Lipid rafts were isolated as described previously by Limpert et al. [56] with some modifications. PCtrk cells (5 × 10^6^) were washed with ice-cold PBS (pH 7.4), then homogenized in 25 mM Tris-HCl, pH 7.5, 150 mM NaCl, 1 mM EDTA (TNE buffer) containing 0.5% Triton X-100 using a Potter-type homogenizer at 4 °C and placed on ice for an additional 30 min. Lysates were then brought up to the same protein concentration. Each sample (1 mL) was placed at the bottom of a discontinuous sucrose gradient by mixing with 3 mL of 2.25 M sucrose in TNE buffer in ultracentrifuge tubes. The resulting sucrose solution was overlaid sequentially with 8.5 mL of 1.2 M and 2.5 mL of 0.15 M sucrose in TNE buffer. Phosphatase and protease inhibitors (Protease inhibitor cocktails; Sigma, USA) were present in all layers. The gradients were spun for 18 h at 60,000× *g* in a Beckman XL-70 centrifuge using an SW32Ti rotor. Subcellular fractions (1 mL each) were collected from the top, yielding 15 fractions in total. The whole procedure was conducted at 4 °C. We also examined the effect of TAK-242 on NTP-induced formation of URFs. PCtrk cells were treated in serum-free DMEM containing saline control, NTP (20 mNU/mL), or NTP and TAK-242 (10 nM) for 3 h, lysed in 0.5% Triton X-100-containing TNE buffer and centrifuged in sucrose density gradients as described above. For Western blotting, equal volumes of each fraction were loaded on SDS-polyacrylamide gels. Quantitation of cholesterol was performed using the Amplex Red Cholesterol Assay Kit (Molecular Probes, Eugene, OR, USA) according to the manufacturer’s instructions. GM1 ± fucosyl GM1 levels were detected by dot blot analysis with cholera toxin subunit B (CTB) conjugated with HRP (CTB-HRP; Molecular Probes) at 1:10,000 as a probe, and quantified by ImageJ software using known concentrations and compositions of a ganglioside mixture obtained from bovine brain (Calbiochem, La Jolla, CA, USA) as a standard. To perform statistical analysis of the Trk protein distribution in lipid rafts and URFs fraction, we measured the densities of each band corresponding to Trk in fractions by ImageJ software. Then, we calculated the ratio of densities of each fraction corresponding to rafts (fraction No. 2–5) and URFs (fraction No. 6–8) against that of fraction No. 1 (this fraction did not contain Trk protein but exhibit background densities) and further calculated the mean densities ± SE of rafts fractions and of URFs. These data were subjected to the statistical analysis (Wilcoxon ranked test). We confirmed that Trk protein is definitely present in lipids rafts fractions in control and NTP-treated cells and NTP-treatment caused a statistically significant redistribution of the Trk protein into URFs fractions than that of control cells.

### 4.6. Statistical Analyses

All data were analyzed after the completion of experiments by SAS software (version 8.2; SAS Institute, Japan). All significance tests used a level <0.05.

## Abbreviations

BDNF	brain-derived neurotrophic factor
DAMPs	damage-associated molecular patterns
NF-M	neurofilament M
NGF	nerve growth factor
NTP	Neurotropin
PRR	pattern recognition receptor
TLR4	toll-like receptor 4
Trk	tropomyosin-related kinase
URFs	unidentified raft-like fractions
